# Predominant Complications of Type 2 Diabetes in Kumasi: A 4-Year Retrospective Cross-Sectional Study at a Teaching Hospital in Ghana

**DOI:** 10.3390/medicina55050125

**Published:** 2019-05-09

**Authors:** Max Efui Annani-Akollor, Otchere Addai-Mensah, Linda Ahenkorah Fondjo, Lorraine Sallah, Eddie-Williams Owiredu, Emmanuel Acheampong, Solomon Akamugri

**Affiliations:** 1 Department of Molecular Medicine, School of Medical Sciences, College of Health Sciences, Kwame Nkrumah University of Science and Technology, PMB, UPO, Kumasi 00233, Ghana; linda.ahenkorahfondjo@yahoo.com (L.A.F.); eddiewilliams.owiredu@gmail.com (E.-W.O.); emmanuelachea1990@yahoo.com (E.A.); 2 Department of Medical Laboratory Technology, Faculty of Allied Health Sciences, College of Health Sciences, Kwame Nkrumah University of Science and Technology, PMB, UPO, Kumasi 00233, Ghana; drmedmozart@yahoo.com (O.A.-M.); solomonakamugri12@gmail.com (S.A.); 3 Department of Physiology, School of Medical Sciences, College of Health Sciences, Kwame Nkrumah University of Science and Technology, PMB, UPO, Kumasi 00233, Ghana; lsallah.chs@knust.edu.gh; 4 School of Medical and Health Science, Edith Cowan University, 270 Joondalup Drive, Joondalup, WA 6027, Australia

**Keywords:** type 2 diabetes mellitus, microvascular, macrovascular, complications

## Abstract

*Background and objectives*: Diabetes mellitus type 2 (T2DM) has been associated with several microvascular and macrovascular complications. However, studies regarding the predominant complications of T2DM in Ghana have not been conducted. This study evaluated the prevalence and predominant complications of T2DM and assessed the sociodemographic factors associated with the development of diabetes-related complications in Kumasi, Ghana. *Materials and Methods*: This was a retrospective cross-sectional study conducted at Komfo Anokye Teaching Hospital (KATH), Kumasi, Ghana. A total of 1600 Ghanaian T2DM adults were included in this study. Patients’ clinical data from 2012 to 2016 were retrieved from the hospital’s archive. *Results*: The prevalence of macrovascular and microvascular complications of T2DM was 31.8% and 35.3% respectively. The prevalence of neuropathy, nephropathy, retinopathy, sexual dysfunction, diabetic keto-acidosis (DKA), and hypoglycemia were 20.8%, 12.5%, 6.5%, 3.8%, 2.0%, and 0.8% respectively. Sexual dysfunction was significantly associated with the male gender compared to females. Being employed: Informal (aOR = 0.479, *p* < 0.0001), and Formal (aOR = 0.475, *p* = 0.0008) was associated with lower age- and sex-adjusted odds of developing T2DM-related complications while having T2DM for 5–10 years (aOR = 1.550, *p* = 0.0009) and more than 10 years (aOR = 2.755, *p* < 0.0001) was associated with increased odds of developing complications. *Conclusions*: Microvascular complication is the most predominant among T2DM in Kumasi, Ghana. The most prevalent T2DM-related microvascular complication in Kumasi, Ghana is neuropathy. Sexual dysfunction is associated with male compared to female T2DM patients. Being employed reduces the chance of developing T2DM-related complications while increasing DM duration increases the risk of complications.

## 1. Introduction

Diabetes mellitus (DM) is a chronic metabolic disorder that assumes global pandemic proportions and accounts for up to 90% of all diagnosed diabetes [1]. Type 2 diabetes mellitus (T2DM) is the most common form of the disease, accounting for approximately 90% of DM cases [1]. The WHO report shows that the prevalence of T2DM is increasing rapidly in developing countries [1]. There is also compelling data showing an increasing incidence and prevalence of T2DM in Africa [2]. According to International Diabetes Federation (IDF), 415 million people have T2DM worldwide with more than 14 million being Africans [3]. In sub-Saharan Africa, it is estimated that 8% of the population above 25 years are T2DM patients [4]. Another report from the IDF showed that a total of 450,000 Ghanaians were living with the disease as in 2014 and this is estimated to reach 820,000 by 2035 with a mortality rate of 8.6% in adults [5]. T2DM occurs as a result of insulin resistance and the inability of the pancreas to increase insulin, and is characterized by chronic hyperglycemia, impaired glucose tolerance, altered insulin secretion and insulin resistance. In addition to its function as a potent predictor of T2DM, insulin resistance is also a therapeutic target in the presence of hyperglycemia among patients with T2DM. Insulin resistance also results in perilous alterations in the vascular endothelium, suppresses lipolysis and results in derangements of lipid profiles, which consequently culminate in cardiovascular morbidities [6,7].

Thus, T2DM, if not properly managed, can result in detrimental complications. These have been categorized into acute and chronic complications. Diabetic ketoacidosis (DKA) and hyperosmolar hyperglycemic state (HHS) are the most life-threatening acute metabolic complications of the disease [6]. The chronic complications of T2DM include macrovascular and microvascular diseases. The microvascular complications include diabetic retinopathy, nephropathy, and peripheral neuropathy, which can lead to blindness, renal failure, and foot ulcer, respectively, as well as sexual dysfunction [7,8]. Coronary heart disease; cerebrovascular disease and peripheral vascular disease are the main macrovascular diseases associated with T2DM [9,10].

T2DM and its complications are a huge burden on the health care system and the national economy. In low-income countries, T2DM patients have a high tendency to experience catastrophic medical spending and often do not possess appropriate medications to treat the condition, thus resulting in diabetes-associated complications [11]. A recent review study reports that the direct cost of T2DM in Africa ranges from 3.5 billion Intl. dollars to 4.5 billion Intl. dollars per annum per country [12]. In Ghana, the average annual financial cost of managing one diabetic case is estimated to be Ghana cedi (GHS) 540.35 (US $372.65), with direct medical cost constituting the highest proportion (78%) of the direct cost while the total financial cost of diabetes management is estimated at GHS 420,087.67 (US $289,715.63) [13]. Development of T2DM alone can sap finances due to the cost of diagnoses, check-ups and treatment [14]. Moreover, the disabilities as a result of complications and the event of premature death due to T2DM can drive a family into impoverishment.

Adequate knowledge of T2DM and its complications is key to the management of the disease in Ghana. Reports show that increasing patients’ knowledge regarding a disease and its complications has significant benefits on patient compliance to treatment and decreasing complications associated with the disease [15,16]. Some research has been done into knowledge and management of the disease in Ghana [17,18]; However, none have been conducted on the complications associated with the disorder. As such, this study retrospectively examined the prevalence and predominant complications of T2DM and evaluated the association between the duration of DM, as well as the sociodemographic factors, with the development of diabetes-related complications at Komfo Anokye Teaching Hospital, Kumasi, Ghana.

## 2. Materials and Methods

### 2.1. Study Design/Area

This was a hospital-based retrospective cross-sectional study at the Diabetic Clinic of the Komfo Anokye Teaching Hospital (KATH), Kumasi, Ghana. KATH is a 1200-bed facility in Kumasi, the capital city of the Ashanti Region-Ghana [19]. It lies between latitude 6.35° N and 6.40° N and longitude 1.3° W and 1.35° W and has a projected population of 4,780,380, and accounting for 19.4% of Ghana’s total population [20]. The diabetic clinic is frequented by more than 100 patients every week [21,22]. Kumasi is the second largest metropolitan area in Ghana. It is in a rainforest region, and is the commercial, industrial and cultural capital of Asanteman.

### 2.2. Study Population and Data Collection

The study involved 1600 type 2 diabetics who were 18 or more years old. Since KATH is a referral center for patients from the upper parts of the country, we included only residents of the Ashanti region. Non-residents and patients below 18 years were excluded. Patients with records of being on insulin injections were also excluded to limit the likelihood of recruiting type 1 diabetic patients. Patients’ clinical data from 2012 to 2016 were retrieved from the hospital’s archive. Data collected include sociodemographic characteristics, duration of diabetes, and reported diabetes complication.

### 2.3. Ethical Considerations

Ethical approval for this study was obtained from the Committee on Human Research Publication and Ethics (CHRPE) of the School of Medical Sciences, Kwame Nkrumah University of Science and Technology (CHRPE/AP/368/17; 30 June 2017) and from the Research and Development Department of KATH.

### 2.4. Definition of Terms

Retinopathy, nephropathy, and neuropathy were considered microvascular complications of diabetes while angina, myocardial infarction, and peripheral artery disease were considered macrovascular (cardiovascular) complications of diabetes in this study.

### 2.5. Data Analysis

All categorical data were presented as frequencies (percentages) and continuous variables as mean ± SD. Chi-squared/Fisher exact test and independent t-test were performed for comparisons of parameters between groups. Multivariate logistic regression analysis was used to assess the socio-demographic risk factors for complication among diabetics. A *p* value <0.05 was considered statistically significant. All statistical analyses were performed using IBM SPSS 25.0 Statistics.

## 3. Results

The mean age of the entire study population was 55.9 years. The overall prevalence of complication among the study population was 59.0%. Most of the study participants were females (60.0%), married (69.3%), employed in the informal sector (76.8%) and had diabetes <5 years (63.5%). Subjects with complication were significantly older compared to those without complication (*p* < 0.001) (Table 1).

**Figure 1:**
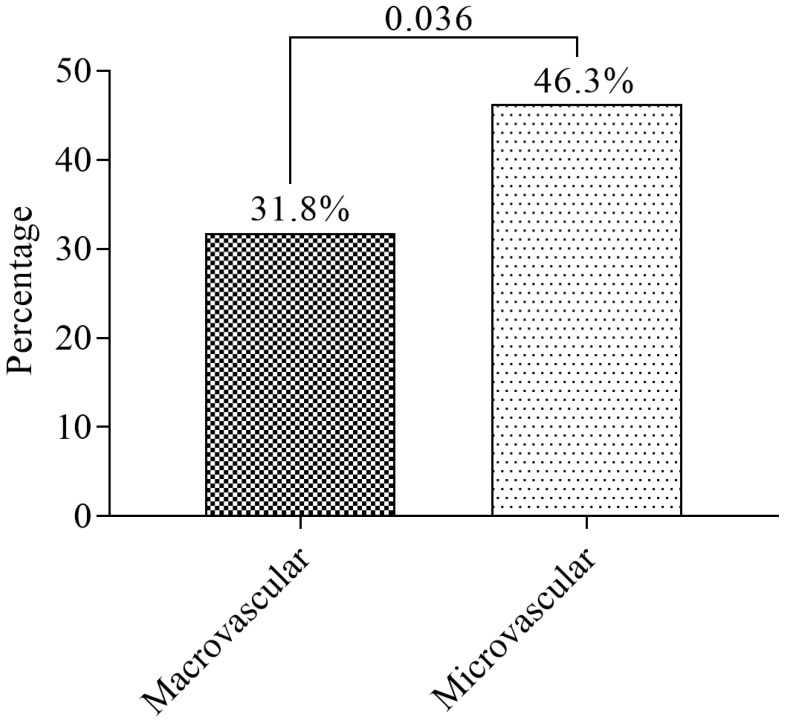
The prevalence of macrovascular and microvascular complications.

The prevalence of macrovascular and microvascular complications of T2DM was 31.8% (508/1600) and 35.3% (564/1600), respectively (Figure 1).

**Figure 2:**
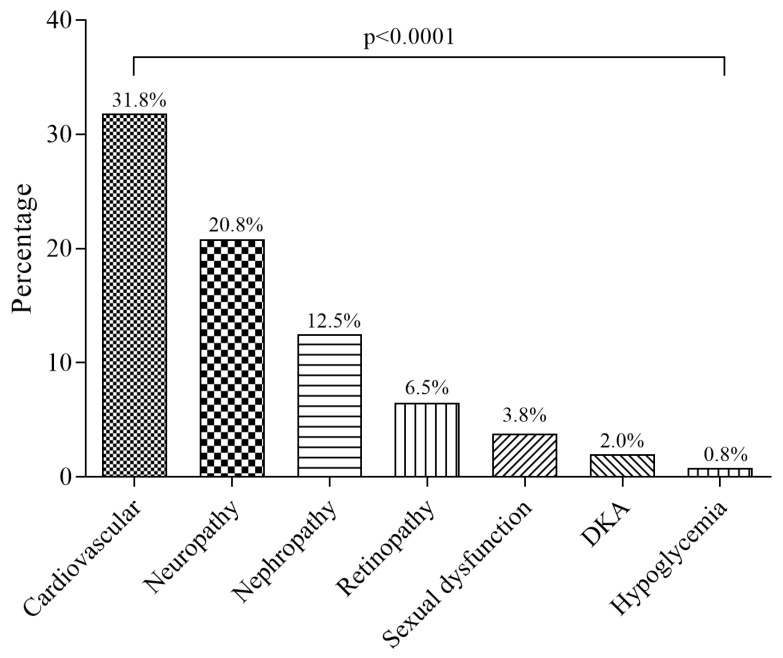
The proportions of individual complications of diabetes among the study participants.

The prevalence of cardiovascular disease (CVD), neuropathy, nephropathy, retinopathy, sexual dysfunction, DKA, and hypoglycemia were 31.8%, 20.8%, 12.5%, 6.5%, 3.8%, 2.0%, and 0.8% respectively (Figure 2).

**Figure 3:**
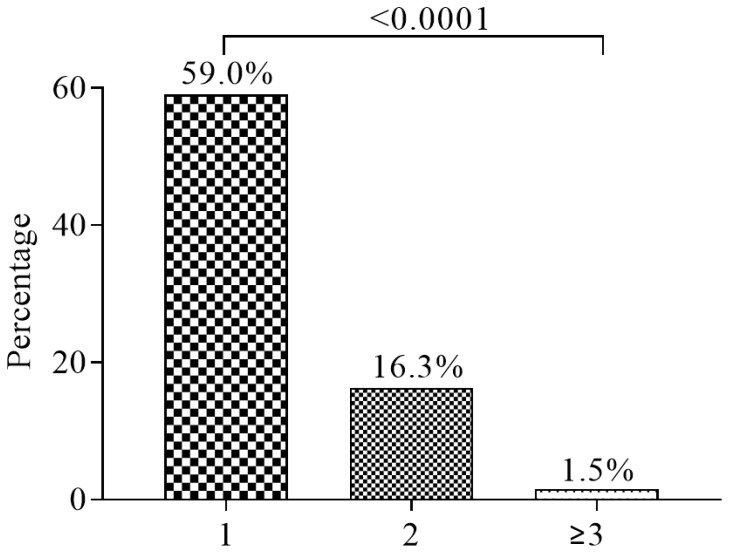
The proportions of participants with single, double and multiple complications of type 2 diabetes mellitus.

The prevalence of single, double, and multiple complications of diabetes were 59.0%, 16.3%, and 1.5%, respectively (Figure 3).

Upon stratifying the complications by gender, most complications were found among females, though sexual dysfunction was significantly associated with the male gender compared to the females (80 vs 20%). In addition, with the exception of nephropathy and hypoglycemia, all other diabetes-related complication was associated with the age group of 51–70 years old and being married. Additionally, subjects with basic educational level was associated with all complications studied except CVD and DKA while subjects working in the informal sector was associated with only retinopathy (Table 2).

In a multivariate logistic regression analysis, to assess the effect of duration of DM and the socio-demographic risk factors associated with complication in diabetes, subjects who were employed: Informal (aOR = 0.479, 95% CI (0.342–0.670), *p* < 0.0001), and Formal (aOR = 0.475, 95% CI (0.307–0.734), *p* = 0.0008) had lower age- and sex-adjusted odds of developing T2DM-related complications. However, having T2DM for 5–10 years (aOR = 1.550, 95% CI (1.196–2.009), *p* = 0.0009) and more than 10 years (aOR = 2.755, 95% CI (2.026–3.746), *p* < 0.0001) was associated with increased odds of developing complications among the study population (Table 3).

**Figure 4:**
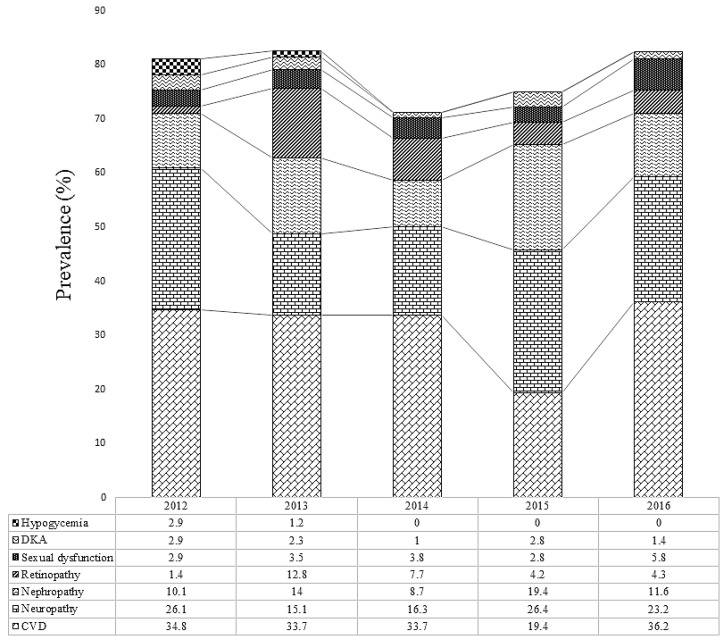
The prevalence of complication of T2DM stratified by years.

CVD was the most prevalent in 2012 (34.8%), 2013 (33.7%), 2014 (33.7%), and 2016 (36.2%). In 2015, neuropathy was the most prevalent complication of diabetes (26.4%) (Figure 4).

## 4. Discussion

The influx of Western culture and lifestyles are increasingly being embraced by African countries with a resultant increase in the prevalence of cardiovascular diseases and diabetes [23]. Type 2 diabetes mellitus (T2DM) patients are particularly predisposed to long-term microvascular and macrovascular complications. The present study presents the prevalence and predominant complications of T2DM and the association between the duration of DM, as well as the sociodemographic factors with the development of diabetes-related complications in Kumasi, Ghana.

This study reports a high prevalence of microvascular and macrovascular (cardiovascular disease) complications of DM (35.3% and 31.8%, respectively). Diabetes-related microvascular complications ensue due to the interplay of metabolic and hemodynamic factors [23] and African diabetics are predominantly predisposed to microvascular complications, partly due to poor compliance, hypertension, poor blood glucose control and the possibility of genetic predisposition [24]. Additionally, though cardiovascular disease (CVD) is considered a disease of Caucasians compared to Africans, evidence suggests an increasing burden of cardiovascular disease in Africa [23]. This finding is in consonance with the finding of a study by Morgan et al. among British diabetics [25] and Shi et al. among Chinese diabetics [26]. Apart from that, some T2DM patients presented with multiple complications (16.3% had two complications and 1.5% had at least three complications), buttressing the need for effective multidimensional approaches to relegate the increasing morbidity due to DM.

Furthermore, the prevalence of neuropathy, nephropathy, retinopathy, sexual dysfunction, DKA, and hypoglycemia in this study were 20.8%, 12.5%, 6.5%, 3.8%, 2%, and 0.8% respectively. This is similar to a cross-sectional study by Yang et al. among Chinese [27], who reported the prevalence of neuropathy, nephropathy, and retinopathy to be 17.8%, and 10.7% respectively. Despite the consistency with the finding of Yang et al., the prevalence of retinopathy observed in their study (14.8%) is higher than in this current study (6.5%); a discrepancy which may be due to the difference in ethnicity or other factors such as diet and lifestyle behaviors. Besides, several epidemiological studies have reported a consistently high prevalence of diabetic retinopathy in China [28,29,30,31]. The findings of this study are also in consonance with a study by Harzallah et al. [32] among diabetic patients over the age of 30 years in Tunisia who reported the prevalence of neuropathy, nephropathy, and retinopathy to be 24%, 13%, and 8% respectively. On the contrary, studies by Macky et al. [33] and Hamed et al. [34], both in Egypt reported retinopathy prevalence of 20% which is higher than the 6.5% observed in this present study. Additionally, Hamed et al. found a higher prevalence of nephropathy (46.3%) and neuropathy (60%) compared to this study. A study by Elbagir et al. [35] also reported a neuropathy prevalence of 36.7% among diabetics in Sudan. Nonetheless, the higher prevalence in their studies could be due to the inclusion of both type 1 and type 2 diabetes while this study included only type 2 DM patients.

This study also found that sexual dysfunction was significantly associated with male T2DM patients compared to their female counterparts; a finding which may be due to hormonal factors as well as occupational and lifestyle practices that predisposes the male gender to poor sexual health. Furthermore, in the assessment of socio-demographic risk factors associated with T2DM-related complications, old age was associated with DM complications. Moreover, participants with complication were significantly older compared to those without complication with majority (55.1%) of the participants who had complications being between 51–70 years old. This finding is consistent with a study by Yang et al. in China [36] who observed that 53.2% of diabetics with complications were within 50–70 years. Additionally, being employed, whether in the formal (aOR = 0.475, 95% CI (0.307–0.734), *p* = 0.0008) or informal sector (aOR = 0.479, 95% CI (0.342–0.670), *p* < 0.0001) was associated with lower age- and sex-adjusted odds of developing T2DM-related complications compared to being unemployed. This could be related to the fact that; unemployment results in financial insecurities and poorer nutritional choices [22] which consequently result in poorer glucose control and reduced overall health status, eventually resulting in complications. Nonetheless, having diabetes for 5–10 years (aOR = 1.550, 95% CI (1.196–2.009), *p* = 0.0009) and more than 10 years (aOR = 2.755, 95% CI (2.026–3.746), *p* < 0.0001) was associated with increased odds of developing T2DM-associated complications among the study population. This is consistent with previous studies including a retrospective, observational study in India by Ramanathan [37], where he found that increasing duration of diabetes was associated with microvascular complications. In addition, a hospital-based study by Macky et al. [33] in Egypt reported that longer duration of DM was associated with diabetic retinopathy. Additionally, a study by Zoungas et al. [38] showed that duration of type 2 diabetes is independently associated with the risk of microvascular and macrovascular complications of diabetes.

When the complications were stratified by years, cardiovascular complication was the most prevalent from 2012 to 2014 and in 2016 with a prevalence rate of 34.8%, 33.7%, 33.7%, and 36.2% respectively. However, in 2015, neuropathy was the most prevalent complication (26.4%). This shows that, the main T2DM-associated complication in Kumasi has been CVD to date; thus, early screening for CVDs among T2DM patients may be valuable in reducing the prevalence of CVD as well as the general complication of DM.

This study is however limited by the fact that the study population was skewed towards the female gender and the age range of 51–70 years old which may have resulted in the increased prevalence among females and subjects within 51–70 years old. Nonetheless, the data obtained provides a possible representation of the distribution of T2DM patients in Kumasi.

## 5. Conclusions

The prevalence of macrovascular and microvascular complications of T2DM in Kumasi is 31.8% and 35.3% respectively. The prevalence cardiovascular disease is 31.8% while the prevalence of neuropathy, nephropathy, retinopathy, sexual dysfunction, diabetic keto-acidosis (DKA), and hypoglycemia are 20.8%, 12.5%, 6.5%, 3.8%, 2.0%, and 0.8% respectively. Generally, microvascular complication is the most prevalent among T2DM in Kumasi, Ghana. The most prevalent T2DM-related microvascular complication in Kumasi, Ghana, is neuropathy. Sexual dysfunction is associated with male compared to female T2DM patients. Being employed reduces the odds of T2DM complications while increasing DM duration increases the odds of developing complications.

## Figures and Tables

**Table 1:** Socio-demographic characteristics of the study participants.

Variables	Total (*n* = 1600)	Complications (944/59.0%)	No Complications (656/41.0%)	*p* value
**Gender**				0.876
Male	640 (40.0)	376 (39.8)	264 (40.2)	
Female	960 (60.0)	568 (60.2)	392 (59.2)	
**Age (years)**	55.9 ± 14.3	58.5 ± 13.6	52.3 ± 14.4	<0.0001
<30	60 (3.8)	32 (3.4)	28 (4.3)	0.001
30–50	484 (30.3)	216 (22.9)	268 (40.8)	
51–70	800 (50.0)	520 (55.1)	280 (42.7)	
≥71	256 (16.0)	176 (18.6)	80 (12.2)	
Gender-wise age ratio (m:f; *p* value)	56.2:56.2; *p* = 0.968	56.7:55.4; *p* = 0.188	57.4:55.6; *p* = 0.105	
**Marital status**				0.012
Single	156 (9.8)	80 (8.5)	76 (11.6)	
Married	1108 (69.3)	644 (68.2)	464 (70.7)	
Widowed	232 (14.5)	156 (16.5)	76 (11.6)	
Divorced	104 (6.5)	64 (6.8)	40 (6.1)	
**Educational status**				0.329
Illiterate	356 (22.3)	216 (22.9)	140 (21.3)	
Basic	640 (40)	360 (38.1)	280 (42.7)	
Secondary	312 (19.5)	192 (20.3)	120 (18.3)	
Tertiary	292 (18.3)	176 (18.6)	116 (17.7)	
**Occupational status**				<0.0001
Informal	1228 (76.8)	700 (74.2)	528 (80.5)	
Formal	176 (11.0)	100 (10.6)	76 (11.6)	
Retired	196 (12.3)	144 (15.3)	52 (7.9)	
**Duration of diabetes (years)**				<0.0001
<5	1016 (63.5)	540 (57.2)	476 (72.2)	
5–10	320 (20.0)	204 (21.2)	116 (17.7)	
>10	264 (16.5)	200 (21.2)	64 (9.7)	

m = male, f = female

**Table 2:** Prevalence of complications stratified by socio-demographic characteristics.

Complications	CVD	Neuropathy	Nephropathy	Retinopathy	Sexual Dysfunction	DKA	Hypoglycemia
**Sex**							
Male	176 (34.6)	144 (43.4)	80 (40.0)	32 (30.8)	48 (80.0) ^‡^	16 (50.0)	0 (0.0)
Female	332 (65.4) ^†^	188 (56.6)	120 (60.0)	72 (69.2)	12 (20.0)	16 (50.0)	12 (100.0) ^‡^
**Age (years)**							
<30	16 (3.1)	28 (8.4)	8 (4.0)	0 (0.0)	4 (6.7)	8 (25.0)	0 (0.0)
30–50	176 (34.6)	96 (28.9)	68 (34.0)	16 (15.4)	12 (20.0)	4 (12.5)	0 (0.0)
51–70	224 (44.1) ^†^	156 (47.0) ^‡^	92 (46.0)	68 (65.4) ‡	40 (66.7) ^†^	16 (50.0) ^‡^	8 (66.7)
>71	92 (18.1)	52 (15.7)	32 (16.0)	20 (19.2)	4 (6.7)	4 (12.5)	4 (33.3)
**Marital status**							
Single	52 (10.2)	40 (12.0)	12 (6.0)	12 (11.5)	4 (6.7)	4 (12.5)	0 (0.0)
Married	340 (66.9)	236 (71.1) ^†^	136 (68.0)	72 (69.2) †	36 (60.0) ^†^	24 (75.0)	12 (100.0)
Divorced	40 (7.9)	16 (4.8)	16 (8.0)	12 (11.5)	16 (26.7)	0 (0.0)	0 (0.0)
Widow	76 (15.0)	40 (12.0)	36 (18.0)	8 (7.7)	4 (6.7)	4 (12.5)	0 (0.0)
**Education**							
None	104 (20.5)	76 (22.9)	44 (22.0)	40 (38.5) ^‡^	8 (13.3)	4 (12.5)	0 (0.0)
Basic	212 (41.7)	124 (37.3) ^†^	100 (50.0) ^‡^	40 (38.5) ^‡^	28 (46.7) ^‡^	16 (50.0)	8 (66.7) ^†^
Secondary	100 (19.7)	56 (16.9)	28 (14.0)	12 (11.5)	20 (33.3)	4 (12.5)	4 (33.3)
Tertiary	92 (18.1)	76 (22.9)	28 (14.0)	12 (11.5)	4 (6.7)	8 (25.0)	0 (0.0)
**Occupation**							
Retired	68 (13.4)	36 (10.8)	20 (10.0)	4 (3.8)	8 (13.3)	4 (12.5)	0 (0.0)
Formal	60 (11.8)	44 (13.3)	20 (10.0)	12 (11.5)	8 (13.3)	4 (12.5)	0 (0.0)
Informal	380 (74.8)	252 (75.9)	160 (80.0)	88 (84.6) ^†^	44 (73.3)	24 (75.0)	12 (100.0)

CVD = Cardiovascular disease, DKA = Diabetic Ketoacidosis; ^†^ Significant at *p* < 0.05, ^‡^ Significant at *p* < 0.01

**Table 3:** Age and gender adjusted multivariable logistic regression analyses of socio-demographic characteristics and duration of diabetes associated with type 2 diabetes mellitus complications.

Variable	aOR (95% CI)	*p* value
**Age (years)**		
<30	1	
30–50	0.705 (0.412–1.208)	0.203
51–70	1.625 (0.959–2.754)	0.071
>71	1.925 (1.087–3.411)	0.025
**Gender**		
Male	1	
Female	1.017 (0.830–1.247)	0.868
**Educational level**		
No education	1	
Basic	0.833 (0.640–1.085)	0.176
Secondary	1.04 (0.759–1.417)	0.819
Tertiary	0.983 (0.717–1.349)	0.917
**Occupational status**		
Retired	1	
Informal	0.479 (0.342–0.670)	<0.0001
Formal	0.475 (0.307–0.734)	0.0008
**Duration of DM (years)**		
<5	1	
5–10	1.550 (1.196–2.009)	0.0009
>10	2.755 (2.026–3.746)	<0.0001
